# Complete Genome Sequence of Fish Pathogen *Aeromonas hydrophila* JBN2301

**DOI:** 10.1128/genomeA.01615-15

**Published:** 2016-01-28

**Authors:** Wuming Yang, Ningqiu Li, Ming Li, Defeng Zhang, Guannan An

**Affiliations:** a School of Animal Science and Nutritional Engineering, Wuhan Polytechnic University, Wuhan, China; b Pearl River Fishery Research Institute, Chinese Academy of Fishery Sciences, Key Laboratory of Fishery Drug Development, Ministry of Agriculture, Key Laboratory of Aquatic Animal Immune Technology, Guangzhou, China

## Abstract

*Aeromonas hydrophila* is one of the most important fish pathogens in China. Here, we report complete genome sequence of a virulent strain, *A. hydrophila* JBN2301, which was isolated from diseased crucian carp.

## GENOME ANNOUNCEMENT

*Aeromonas hydrophila*, belonging to the family *Aeromonadaceae*, is an autochthonous species in freshwater environments and a member of the normal microflora in the fish intestinal tract (1). However, under stress conditions, some virulent strains of *A. hydrophila* can invade the most majority of freshwater fish species and infect them with hemorrhagic septicemia. In China, the fish disease caused by this microorganism has become the most important bacterial disease of fish to date and leads to great economic losses periodically per year (2–4). In July 2009, an epidemic septicemia with continuous fish mortality broke out in a crucian carp pond of Guanqiao Fishery surrounded by Donghu Lake, Wuhan, China. *A. hydrophila* JBN2301, isolated from a diseased crucian carp, is highly virulent to crucian carp and zebrafish, with a 50% lethal dose (LD_50_) of <3 × 10^5^ CFU per fish.

Whole-genome sequencing was performed employing Illumina genome analyzer (BGI, Shenzhen, China) using a shotgun strategy, which produced paired-ends totaling about 724 Mb with about 180-fold coverage of the genome. Genome sequence data were processed and assembled into 28 contigs and 6 scaffolds with the software SOAP*denovo* version 2.04 (5). Gaps between contigs were closed by combinatorial PCR and sequencing amplicons by primer walking. Finally, this assembly process produced a complete genome with one circular chromosome and three circular plasmids.

Gene prediction was performed using the NCBI Prokaryotic Genome Annotation Pipeline (2013 release). The genome of strain JBN2301 includes a chromosome with a length of 5,127,362 bp and three plasmids with lengths of 6,318 bp, 6,162 bp, and 6,045 bp. This genome contains 4,438 protein-coding genes, with a G+C content of 60.77%. A total of 129 tRNA genes were predicted by tRNAscan-SE (6), while 10 rRNA operons were predicted by RNAmmer (7).

The *A. hydrophila* JBN2301 genome was also annotated by RAST (8) to facilitate a comparison with *A. hydrophila* ATCC 7966. Among 95 genes involving virulence, disease, and defense, there are two genes involving bile hydrolysis and lysozyme inhibitor in strain JBN2301 only, which enables this strain to survive in the fish immune system. Compared to ATCC 7966, JBN2301 uniquely encodes 17 proteins, which are entirely associated with bacteriophages.

The plasmids from *A. hydrophila* JBN2301 have unique features. Plasmid1 (6,318 bp) contains five genes, including those encoding the YdcE-YdcD toxin-antitoxin system, whose role is to prevent programming death of host cells. Plasmid2 (6,162 bp) carries seven genes, including those encoding ferric enterobactin uptake protein FepE and the toxin-antitoxin replicon stabilization system. Plasmid3 (6,045 bp) contains genes encoding three hypothetical proteins with unknown functions.

In summary, the genome sequence of *A. hydrophila* JBN2301 contributes to the understanding of its mechanism of survival in the pathogen-host interaction environment and plasmid-carried genes encoding the toxin-antitoxin system. Further comparison with the genomes of many virulent and avirulent strains will help search for conserved virulence genes of this bacterial pathogen.

### Nucleotide sequence accession numbers.

The complete genome sequence of *A. hydrophila* JBN2301 was deposited at DDBJ/EMBL/GenBank under the accession numbers CP013178 to CP013181.
